# Positive effects of *Cordyceps cateniannulata* colonization in tobacco: Growth promotion and resistance to abiotic stress

**DOI:** 10.3389/fmicb.2023.1131184

**Published:** 2023-04-14

**Authors:** Lu Qiao, Jing Liu, Zhengxiong Zhou, Zhimo Li, Yeming Zhou, Shaohuan Xu, Zhengkai Yang, Jiaojiao Qu, Xiao Zou

**Affiliations:** ^1^Institute of Fungus Resources, College of Life Sciences, Guizhou University, Guiyang, China; ^2^Zunyi Tobacco Company of Guizhou Province, Zunyi, China; ^3^College of Tea Sciences, Guizhou University, Guiyang, China

**Keywords:** *Cordyceps cateniannulata*, tobacco, endophytic colonization, growing development, rhizosphere microbial diversity

## Abstract

**Background:**

Entomopathogenic fungi can live in insects to cause disease and death and are the largest group of entomopathogenic microorganisms. Therefore, these fungi are best known for their microbial control potential. Importantly, they also have other beneficial effects, including promoting plant growth and development by colonizing plant. Here, the study sought to identify specific strains of the entomopathogenic fungus, *Cordyceps cateniannulata* that would form endophytic associations with tobacco, thus benefiting plant growth and resistance to abiotic stresses, thereby highlighting the application of entomopathogenic fungi in tobacco.

**Methods:**

The *C. cateniannulata*-tobacco symbiont was constructed by root irrigation. The effects of *C. cateniannulata* on tobacco growth were evaluated by measuring the maximum leaf length, maximum leaf width, number of leaves, plant height, stem thickness, stem circumference, dry and fresh shoot weight 7, 14, 21, and 28 days after colonization. The peroxidase, catalase, superoxide dismutase, and malondialdehyde were measured to observe the impact of *C. cateniannulata* on tobacco defense enzyme activity. Finally, high-throughput sequencing was used to access microbial communities in the rhizosphere, with data subsequently linked to growth indicators.

**Results:**

After tobacco was inoculated with *C. cateniannulata* X8, which significantly promoted growth and related enzyme activity, malondialdehyde was decreased. The most significant impact was on peroxidase, with its activity being upregulated by 98.20, 154.42, 180.65, and 170.38% in the four time periods, respectively. The high throughput sequencing results indicated that *C. cateniannulata* had changed the rhizosphere microbial relative abundances, such as increasing Acidobacteria and Ascomycetes, and decreasing Actinomycetes and Basidiomycetes. The redundancy analysis showed that *C. cateniannulata* significantly boosted tobacco growth by reducing the abundance of specific dominant genera such as *Stachybotrys*, *Cephalotrichum*, *Streptomyces*, *Isoptericola*, and *Microbacterium*.

**Conclusion:**

Specific strains of *C. cateniannulata* can be introduced into host plants as endophytes, resulting in promotion of host plant growth and increased resistance to abiotic stress and microbial pathogens. The study provides a foundation for future studies of *C. cateniannulata* as an ecological agent.

## 1. Introduction

Entomopathogenic fungi are direct sources of fungal insecticides and refer to fungi that live in insects to cause disease and death, thus they are best known for their microbial control potential (Raya-Díaz et al., 2017; Shin et al., 2020). Studies have shown that some entomopathogenic fungi have dual hosts in nature (Lacey et al., 2015). These fungi can parasitize insects, as well as colonize plants to interact with plants without causing harm (Barelli et al., 2016). As such, they have gradually been included in the category of plant endophytes (González-Guzmán et al., 2022; Liu et al., 2022). Some scholars have found that entomopathogenic fungi *Beauveria bassiana* and *Metarhizium brunneum* can be used as plant endophytes, promoting the growth of wheat, maize, and broad bean (Jaber, 2018; Russo et al., 2019; Rasool et al., 2021). *M. anisopliae* transfers nitrogen from insects to host plants through fungal hyphae to promote plant growth and development (Behie et al., 2012).

Abiotic stress is the most harmful factor affecting crop yields. Plants have developed a set of antioxidant defense mechanisms in response to abiotic stress through defensive enzymes, such as peroxidase (POD), superoxide dismutase (SOD), and catalase (CAT) (Tang et al., 2019), which constitute the antioxidant enzyme-promoting system in living organisms (Zhou et al., 2021). Malondialdehyde (MDA) is a membrane lipid peroxidation product that reflects the degree of cell damage (Xu et al., 2013). Low concentrations of MDA are conducive to the growth and resistance of symbiotic plants to environmental stress (Khan et al., 2015). Entomopathogenic fungi can maintain plant growth by increasing the activity of antioxidant enzymes and decreasing MDA expression, thereby alleviating the damage caused by environmental stress (Tsikas, 2017; González-Pérez et al., 2022). Researchers have discovered that several key defense enzyme activities, including POD, SOD, and CAT are enhanced in maize seedlings after colonizing *B. bassiana* (Forlani et al., 2014). Entomopathogenic fungi provides a basis for the application of the concept of ecological symbiosis in plant protection.

The entomopathogenic fungus *Cordyceps cateniannulata*, also known as *Paecilomyces cateniannulatus*, belongs to the Sordariomycetes, Hypocreales, and Cordycipitaceae families (Zhang et al., 2016). As an essential group of broad-spectrum entomopathogenic fungi, *C. cateniannulata* plays a vital role in plant pest control, with the fatality rate to tetranychus being as high as 92% (Zhang et al., 2018). Most studies have focused on the application of *C. cateniannulata* in biological control. In addition, this fungus also acts to promote the growth of host plants. In recent years, reports on growth promotion have gradually emerged, such as it could colonize and promote the growth of buckwheat (Zhang et al., 2022), increasing the germination index of seeds of buckwheat (Peng et al., 2022), and increasing the tomato biomass (Guan et al., 2022). However, the holistic evaluation of *C. cateniannulata* on the plant and the effect on the rhizosphere soil has not been explored yet.

Tobacco as an essential cash crop, with growth index and yield that are closely related to economic value (Ren et al., 2021). However, tobacco has a great demand for chemical fertilizer and is susceptible to abiotic stress, such as waterlogging, resulting in reduced production and quality, especially in the rainy south (Pan et al., 2019). Therefore, to find and develop an efficient and ecological plant protection technology has become an important task for the sustainable development of tobacco industry. Some studies have suggested that *C. cateniannulata* colonizes tomato seedling roots, stems, and leaves by immersion, which may promote plant growth and antioxidant enzyme activity (Guan et al., 2022). Both tobacco and tomatoes are Solanaceae crops, which begs the question–can *C. cateniannulata* also colonize tobacco? How does *C. cateniannulata* affect tobacco growth development and rhizosphere microbial diversity post-colonization? However, these are open questions. Therefore, the objectives of this study were: (i) to assess the effect of *C. cateniannulata* on plant development by measuring the growth and enzymatic activity of tobacco; (ii) to evaluate the correlation between the dominant microbial genus and tobacco growth; (iii) to provide strain resources for developing a kind of environmental protection biological agent in tobacco.

## 2. Materials and methods

### 2.1. Materials

The strain *C. cateniannulata* GZUIFR04XS8 (X8) was collected from the Institute of Fungal Resources, Guizhou University (GZUIFR), Guiyang, Guizhou, China. The tobacco variety was K326, provided by Guizhou Provincial Key Laboratory of Tobacco Quality Research. POD, SOD, CAT, and MDA enzyme activity detection kits were purchased from Solarbio Technology Co., LTD (Beijing, China). The Cu^2+^ medium formulation included Glucose 40 g, peptone 10 g, agar 18 g, CuSO_4_·5H_2_O powder 1 g, streptomycin sulfate 0.1 g, penicillin potassium 0.1 g, and water 1,000 ml, sterilized at 121°C for 30 min. Seedling trays (50 holes, each 5 cm × 5 cm × 5 cm).

### 2.2. Detection of *Cordyceps cateniannulata* colonization in tobacco

The strain was cultured on potato dextrose agar (PDA) plates at 26°C for 10 d under 12-h light/12-h dark conditions. Conidia were harvested by flooding the plate with sterile 0.05% (v/v) Tween-80 solution. The conidial suspensions were vortexed for 1 min and filtered through two layers of lens paper, with conidia concentrations being measured on a hemocytometer and adjusted to 2 × 10^7^ conidia mL^−1^ using sterile water.

Tobacco seeds were disinfected by sequentially treating them with 75% (v/v) ethanol for 30 s, 10% (v/v) NaClO for 5 min, and sterile water for 1 min. Two seeds were added per individual hole in a seedling tray, filled with sterilized soil. The trays were stored in a sterile artificial climate chamber at 26°C with 70% relative humidity and a 12-h light/12-h dark cycle. The nutrient solution was used to water plants every 3 days.

After 15 days, the tobacco was irrigated with 15 ml of the prepared *C. cateniannulata* conidia suspension on roots for the colonization detection experiments. Upper portions of plants were sampled at 7 days after inoculation, then disinfected (method of disinfection as above), and taking 100 μl of the last clean sterile water for culture to test the disinfection effect of the plant surface. Based on the tolerance of *C. cateniannulata* to Cu^2+^, so the Cu^2+^ medium was used to isolate and culture *C. cateniannulata*. Tissues of roots, stems, and leaves were cut into 1 cm pieces (roots and stems) or 1 × 1 cm (leaves) on a sterile operating table. Respectively, nine pieces were then randomly selected and cultured on Cu^2+^ medium at 26°C until they grew out fungi for use (Xu, 2020).

### 2.3. Microscopic morphology and molecular identification of *Cordyceps cateniannulata* in tobacco

The induced fungal colonies were inoculated on PDA at 26°C for 5 d under 12-h light/12-h dark conditions, Relative Humidity (RH) = 70%. Fresh hyphae were stained with lactophenol cotton blue solution and then observed using an optical microscope. All extracted DNA samples were evaluated by PCR amplification. The internal transcribed spacer (ITS) was amplified using the ITS1 (5 ‘-TCCGTAGGTGAACCTGCGG-3’) and ITS4 (5 ‘-TCCTCCGCTTATTGATATGC-3’) primer pair (White et al., 1990). The PCR reaction system (25 μl) included 2 μl gDNA, 12.5 μl 2 × A8 PCR master mix, 1 μl (10 μmol/l) ITS1, 4, respectively, and ddH_2_O was added to 25 μl (Chiriví-Salomón et al., 2015). The PCR amplification protocol began at 95°C for 3 min, 55°C for 30 s, 72°C for 45 s, followed by 30 cycles and 5 min extension at 72°C (Zhou et al., 2018). The PCR products were monitored by electrophoresis on a 2% (w/v) agarose gel and sequenced by Tsingke Biotechnology Co., LTD (Shanghai, China). A basic local alignment search tool (BLAST) search was performed against DNA sequences in GenBank, and the sequences of related species (similarity>85%) were downloaded. Using *Purpureocillium lilacinum* and *Torrubiella ratticaudata* as the outgroup, the neighbor-joining method constructed a phylogenetic tree using MEGA 7 software with 1,000 bootstrap replicates.

### 2.4. Impact of *Cordyceps cateniannulata* on tobacco growth

Two seeds were added to individual holes in a seedling tray filled with soil, after which all were stored in a natural condition (all materials were not sterilized). Four hundred seeds were planted in four trays (2 seeds/holes × 50 holes × 4 trays). The nutrient solution was used to water plants every 3 days. The seedlings were transplanted to 12-cm diameter pots after 15 days, and one group was designated as the treatment group and irrigated with 15 ml of the prepared *C. cateniannulata* conidia suspension on roots, while the other group was maintained as the control group and irrigated with 15 ml of 0.05% (v/v) Tween-80 solution. The growth indicators of tobacco were measured at 7, 14, 21, and 28 days after inoculation, including maximum leaf length, maximum leaf width, plant height, number of leaves, stem thickness, stem circumference, dry and fresh shoot weight. The maximum leaf length and width were selected from the longest and widest leaves of the tobacco plants and measured with a ruler. The height of the plant was measured cm using a tape measure. The number of leaves was counted from the lower part of the tobacco plant upwards, avoiding the two leaves at the bottom and the two young true leaves facing the top. The stem circumference was measured with a soft ruler about 2 cm from the ground near the plant. The stem thickness was measured by a vernier caliper. Above-ground sections were separated, fresh weights were determined using an analytical balance, and dry weights were examined after drying at 110°C for 2 h, followed by 37°C for 4 days. Five randomly sampled seedlings were used for all experiments.

### 2.5. Impact of *Cordyceps cateniannulata* on tobacco defense enzyme activity

The tobacco in section 2.4 was also used for enzyme activity detection. In both the control and treated groups, mature leaves of 20 plants, each at the same position (middle part of the tobacco stem) were collected to determine POD, SOD, CAT, and MDA content 7, 14, 21 and 28 days after inoculation. Next, 0.1 g fresh leaves were added to 2 ml of phosphoric acid buffer in a pre-cooled mortar and ground with liquid nitrogen until homogenized. Samples were then spun for 20 min at 8000 rpm at 4°C. Supernatants were used as the enzyme extract to determine the enzyme activities. All extraction procedures were conducted based on protocols provided in a kit obtained from Solarbio.

### 2.6. DNA extraction, PCR amplification, and sequencing of soil

The tobacco soil was collected using sterile gloves and placed in sterile plastic bags. The microbial genomic DNA used for high-throughput sequencing of soil was extracted using the FastDNA® SPIN Kit for Soil. The fungal ITS gene was amplified with the primers ITS1F (5′-CTTGGTCATT TAGAGGAAGTAA-3′) and 2043R (5′-GCTGCGTTCTTCATCGATGC-3′). The primers 338F (5′-ACTCCTACGGGAGGCAGCAG-3′) and 806R (5′- GGACTACHVGGGTWTCTAAT-3′) were used to amplify the V3–4 variable region of the bacterial 16S rRNA gene (Adams et al., 2013). All PCR reactions were performed using Phusion® High-Fidelity PCR Master Mix (New England Biolabs). The amplification products were monitored by electrophoresis on 2% (w/v) agarose gel and sequenced using the Illumina HiSeq platform (Majorbio Bio-pharm Technology Co., Ltd., Shanghai, China).

Raw reads from original DNA fragments were merged and quality-filtered using FLASH version 1.2.7 and Trimmomatic version 0.32. The UPARSE version 7.1 was used for chimera removal. Bacterial gene sequences were annotated with taxonomic information using the RDP classifier against the Silva 16S rRNA database. The OTU taxonomic information of fungi was obtained by aligning representative sequences against the Unit 8.0 ITS database (Rognes et al., 2016).

### 2.7. Data analysis

A one-way analysis of variance (ANOVA) followed by least significant difference (LSD) test at *p* < 0.05 was performed using SPSS 25.0 software to detect significant differences among treatments. *p* values <0.05 were considered statistically significant. The alpha diversity of the microbial communities was calculated using the QIIME platform. Analyzes based on the Shannon diversity index and Ace index were performed. The correlation between tobacco growth indicators and dominant soil genera was analyzed on the Metagenomics Core Microbiome Exploration Tool (MetaCoMET).

## 3. Results

### 3.1. Colonization of *Cordyceps cateniannulata* in tobacco

**Figure 1:**
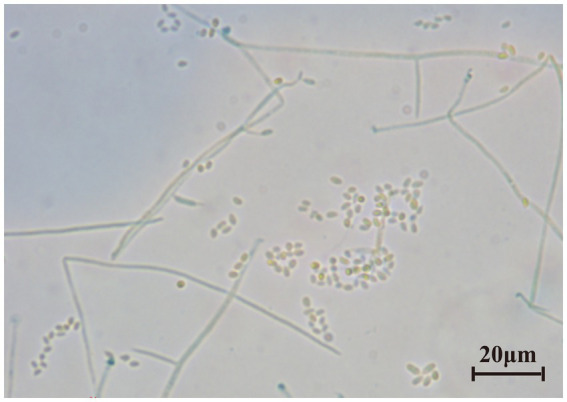
The microscopic morphology of tobacco-isolated strain. Scale bar = 20 μm.

**Figure 2:**
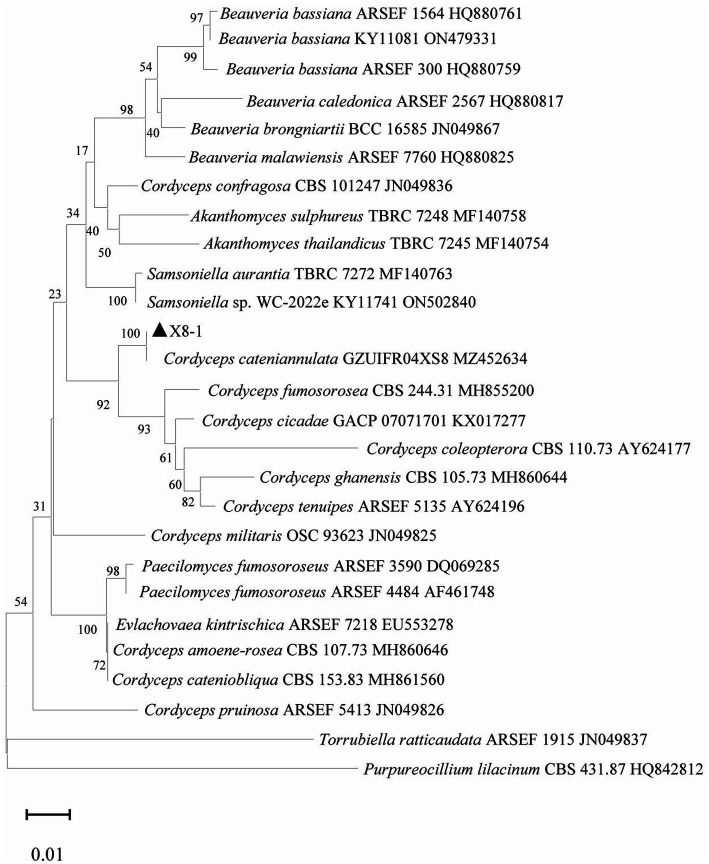
Phylogenetic tree generated with rDNA-ITS sequences of strain X and other related species.

The last water used for washing tissues was cultured, and no colonies formed on the PDA after 5 days, indicating that the sample’s surface had been thoroughly disinfected. The obtained endophytic strain isolated from plant was named X, and colony color, conidial stem, and conidial morphology were all consistent with *C. cateniannulata* (Figure 1; Table 1). The phylogenetic tree based on rDNA-ITS showed that strain X was in the same branch as *C. cateniannulata* GZUIFR04XS8, with 100% similarity (Figure 2).

**Table 1:** Morphological comparison between strain X and *C. cateniannulata.*

Strain	Colonies	Conidiogenous cell	Conidia	Reference
*C. cateniannulata*	In white, raised, and cotton-like. Bottom side pale yellow	In whorls, short, about 13 ~ 24 μm, spherical at the base, tapered upwards at 1/2 of the total length, 4.5 ~ 10 × 1.5 ~ 3 μm	In transparent, smooth, mostly oval or nearly spherical, 2 ~ 4.5 × 1.5 ~ 2 μm, forming shingles arranged oblique chains or rings	Wang et al. (2014)
X	In white, partly yellowish, circular, hyphae tightly connected. Bottom side yellowish-white	In whorls, about 13 μm, most of the peduncles spherically expanded 1.8 ~ 4.5 × 1.2 ~ 2.1 μm at the base, tapered upward at 1/2 of the total length 3.1 ~ 4.3 × 0.9 ~ 1.6 μm	In transparent, smooth, ovoid, or ellipsoidal, about 2.4× 1.2 μm, often with imbricated oblique chains or rings	This paper

### 3.2. The impact of *Cordyceps cateniannulata* on tobacco growth

**Figure 3:**
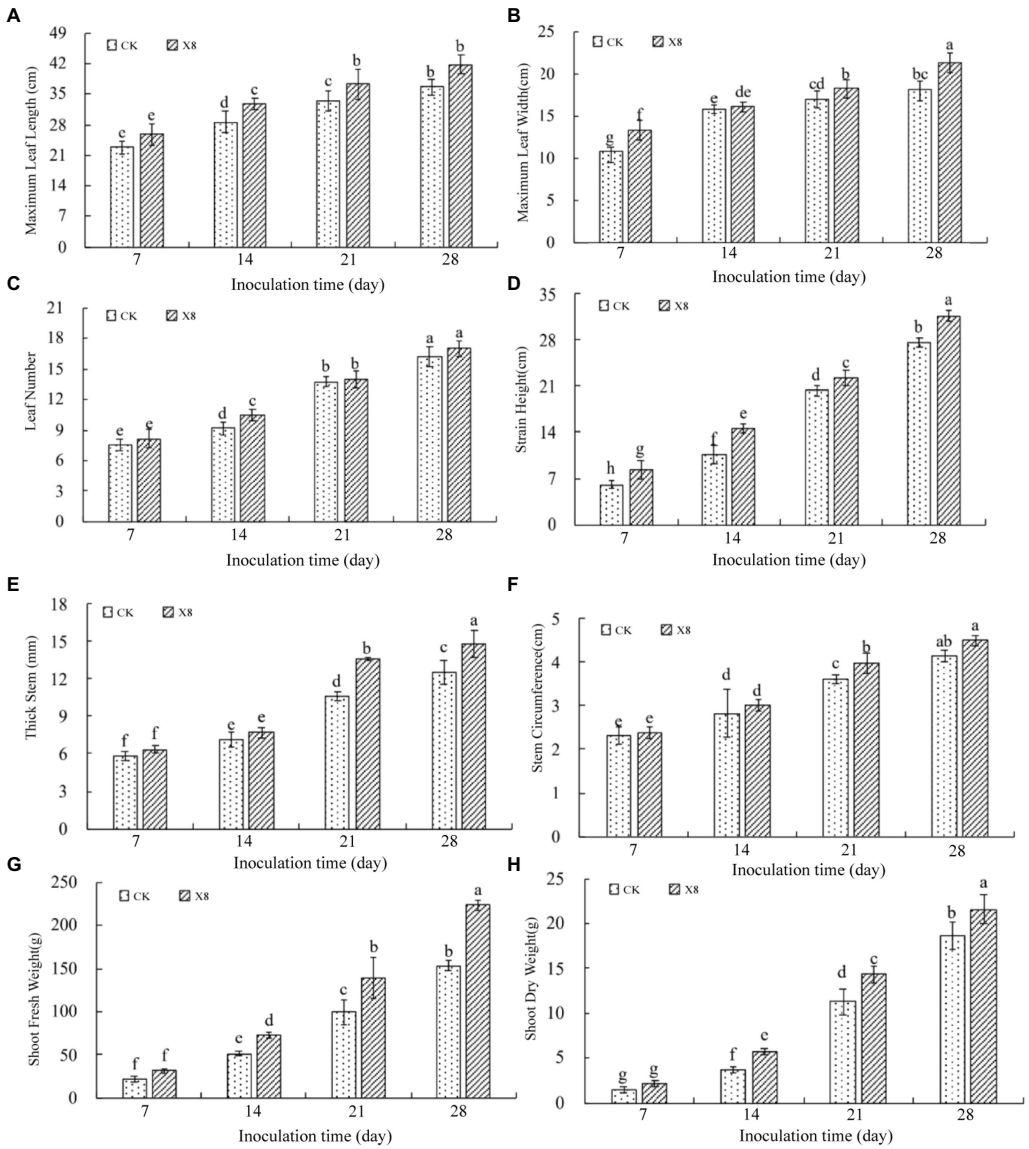
Impact of X8 on tobacco growth indicators. The error bars reflect standard error, mean (±SE) (*n* = 3). Lowercase letters refer to significant differences (LSD test, *p* < 0.05). **(A)** Maximum leaf length, **(B)** maximum leaf width, **(C)** number of leaves, **(D)** plant height, **(E)** stem thickness, **(F)** stem circumference, **(G)** fresh weight, **(H)** dry weight.

The growth and development of tobacco seedlings in the X8 group were superior to those in the control group. The maximum leaf length and width treated with X8 was larger than the control group in the four periods after inoculation. The maximum leaf length reached a significant level on day 14 and 21 (*p* < 0.05), which were 14.69 and 11.41% higher than the control, respectively (Figure 3A). The maximum leaf width reached a significant level on day 7 and 28 (*p* < 0.05), which were 23.72 and 18.13% higher than the control, respectively (Figure 3B). The X8 group had more leaves than the control group, which was 14.88% higher on day 14 (*p* < 0.05; Figure 3C). The plant height of the X8 treatment was significantly higher than the control (*p* < 0.05), which was 37.92% higher and reached a significant difference in the mid-term after transplanting (Figure 3D).

Similarly, the stem thickness and circumference of the X8 treatment group were larger than the control, especially in the last two periods (*p* < 0.05). On day 21 and 28, the stem thickness was 28.46 and 18.41% higher than the control group (Figure 3E), and the stem circumference was 9.64 and 8.45% higher than the control, respectively (Figure 3F).

The dry and fresh shoot weight in the X8 treatment group was greater than the control group, but this was not significant on day 7 after transplanting. However, the difference reached a significant level in the last three periods (*p* < 0.05). The fresh weight in the X8 treatment group was 42.14, 40.38, and 46.26% higher than the control group (Figure 3G). The dry weight was 57.65, 26.96, and 15.92% higher than the control group, respectively (Figure 3H). The results indicated that X8 inoculation was beneficial for accumulating fresh and dry weight in the tobacco.

### 3.3. Impact of *Cordyceps cateniannulata* on tobacco defense enzyme activity

**Figure 4:**
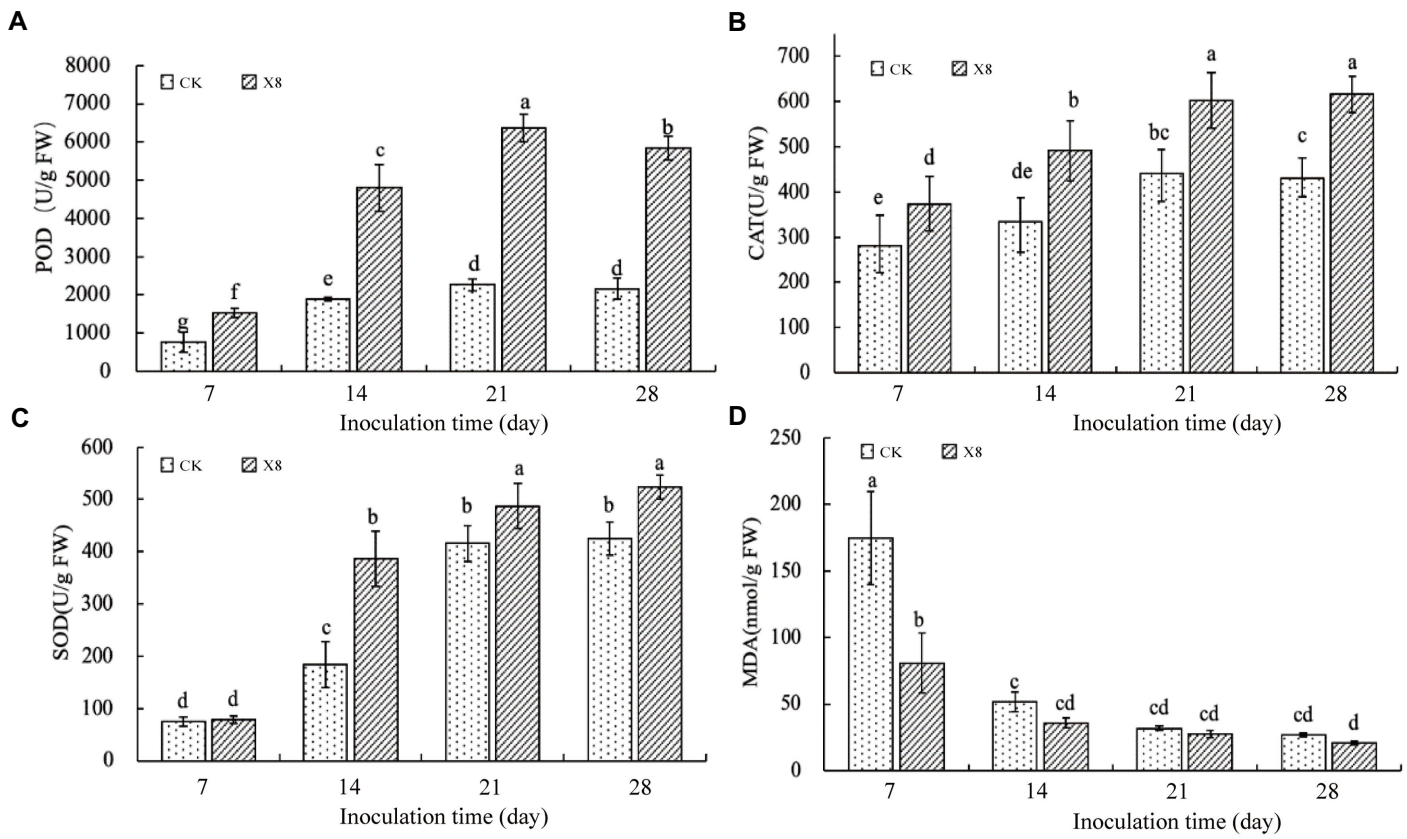
Impact of X8 on tobacco defense enzyme activities. The error bars reflect standard error, mean (±SE) (*n* = 3). The different lowercase letters refer to significant differences (LSD test, *p* < 0.05). **(A)** POD, **(B)** CAT, **(C)** SOD, **(D)** MDA.

The experimental results showed that the X8 treatment significantly increased the activity of antioxidant enzymes (*p* < 0.05) and decreased MDA content. The activity of POD in the X8 treatment group was significantly higher than the control group after transplanting (*p* < 0.05), which was 180.65 and 170.38% higher than the control group after 21 and 28 days, respectively (Figure 4A). The CAT activity of the X8 treatment group was higher than the control group. The differences between the two groups was significant in the last three periods (*p* < 0.05), which was 47.44, 36.72, and 43.35% higher, respectively (Figure 4B). The SOD activity in the X8 treatment group was 4.28, 111.09, 17.15, and 22.92% higher than the control group, respectively, the later stages reached significant levels (*p* < 0.05; Figure 4C). The results showed that X8 inoculation was beneficial for increasing POD, CAT, and SOD activity in tobacco.

MDA content represents the peroxidation degree of the plant cell membrane (Tsikas, 2017). The MDA content in tobacco decreased gradually with the increase of time (Figure 4D). The sampled tobacco had the highest MDA content in the first 7 days, and X8 effectively reduced the content by 116.13% compared to the control. The X8 group had downregulated MDA expression than the control group in the last three periods, but this was not significant, suggesting that the reduction in MDA was most pronounced at the early stage after X8 inoculation.

### 3.4. Analysis of microbial diversities in the rhizosphere

**Figure 5:**
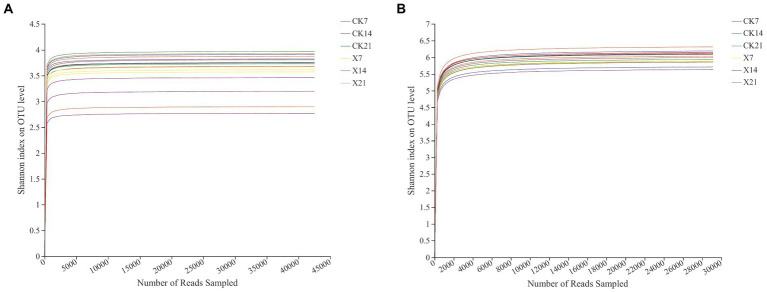
Shannon-Winner index curve. **(A)** Fungal community dilution curve. **(B)** Bacterial community dilution curve.

With the increase of the sample number, the Shannon-Winner index curve tended to be flatter (Figure 5), indicating that the sequencing data sufficiently reflected the microorganisms in the sequencing samples fully. The coverage indices of bacterial and fungal samples were above 98%, indicating that the sample numbers were sufficient to reflect the microbial diversities.

**Figure 6:**
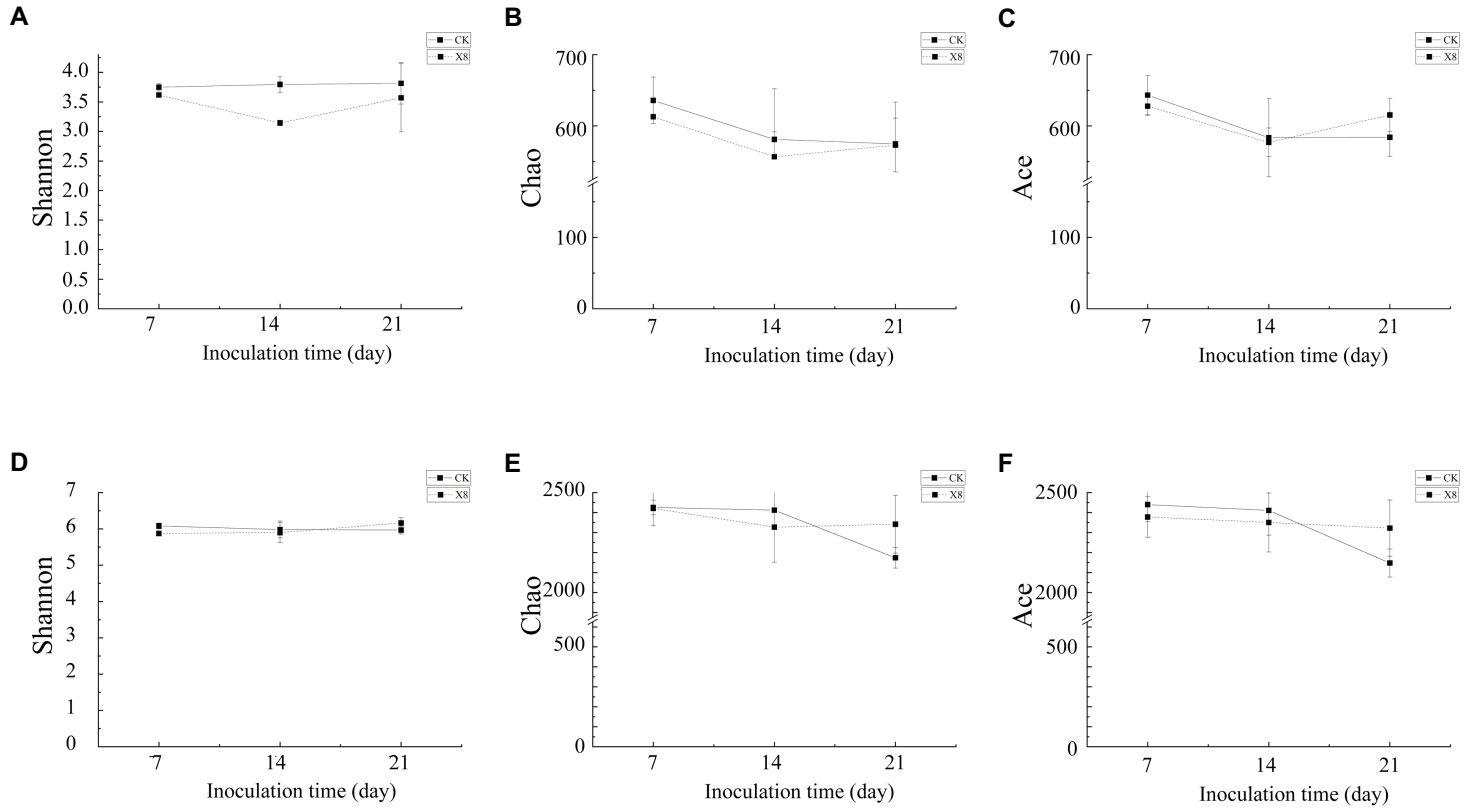
Alpha diversity of fungal and bacterial communities. **(A–C)** Fungal community. **(D–F)** Bacterial community. X8 refers to *C. cateniannulata* X8, CK refers to the control. All the data are presented as mean ± SE (*n* = 3). SE–standard error of the mean.

The Shannon and Chao index of fungi in the X8 treatment group was lower than the control group, and the Ace index was higher than the control group on day 21. The Shannon, Ace, and Chao indexes of bacteria in the X8 treatment group were lower than the control group in the first two periods, and higher than the control group in the later periods. In general, the microbial diversity and richness in the *C. cateniannulata* treatment group increased first and then decreased with time (Figure 6).

### 3.5. Analysis of microbial community composition

**Figure 7:**
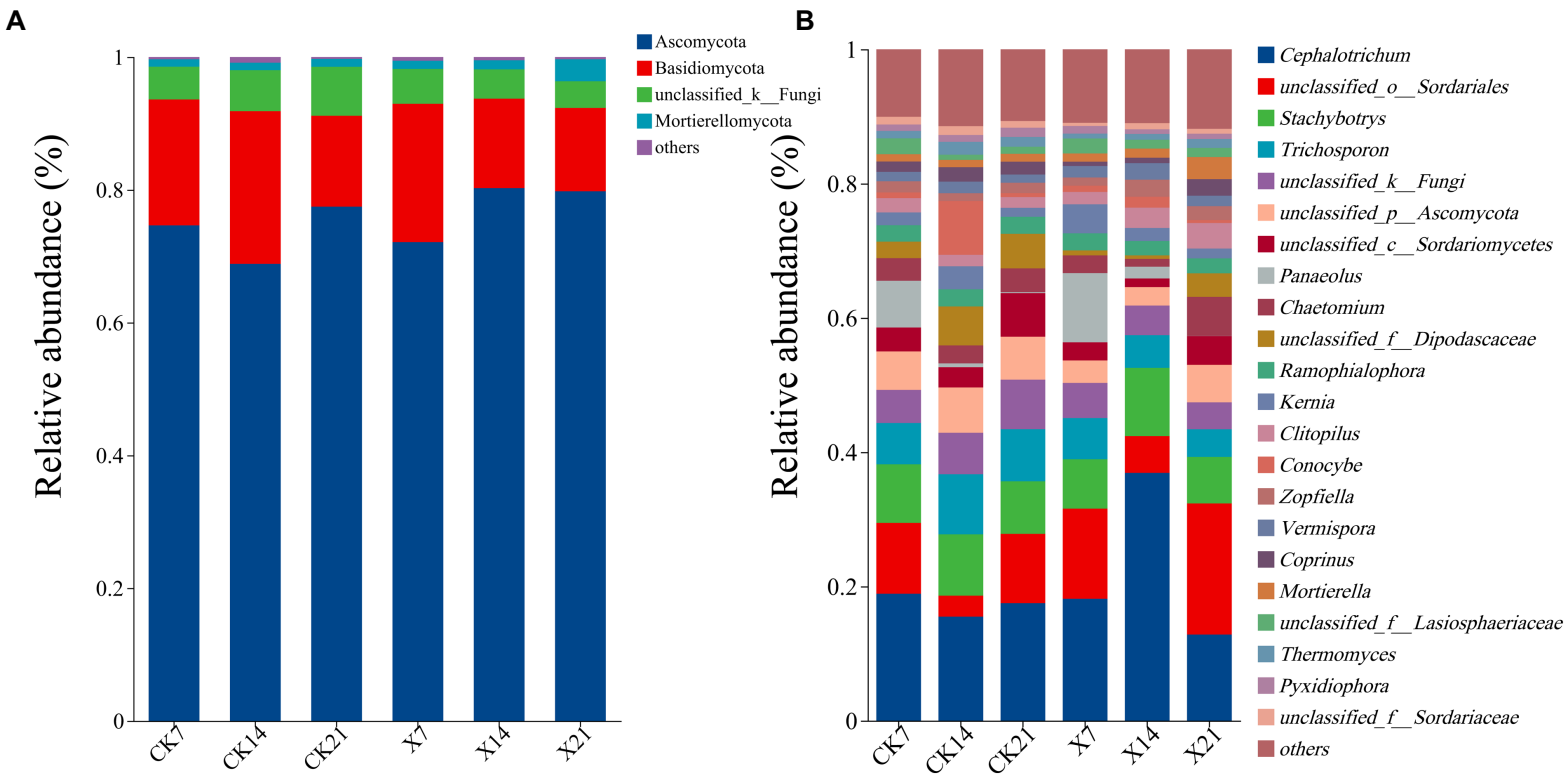
Fungal flora composition at phylum and genus levels. **(A)** Phylum level. **(B)** Genus level, genera with relative abundance less than 1% are classified as other. X refers to *C. cateniannulata* X8 treatment, CK refers to the control, and the number indicates the time point after inoculation.

Species analysis of the fungal community revealed that there were 1,036,450 valid sequences, including 15 phyla, 40 classes, 80 orders, 168 families, 314 genera, 510 species, and 1,120 OTUs. At the phylum level (Figure 7A), Ascomycota was the dominant phylum in all periods of the two groups, with a relative abundance of 68.86–80.26%. The sub-dominant phylum was Basidiomycota, with a relative abundance of 12.55–20.84%. The percentages of Ascomycota and Basidiomycota communities in the X8 group did not change much on day 7 and 21, but this increased and decreased on day 14, respectively. At the genus level (Figure 7B), the dominant genus was *Cephalotrichum* (12.84–36.93%). Compared with the control group, the proportion of *Cephalotrichum* in the X8 group decreased first, then increased, and its abundance increased significantly on day 14. The abundance of *Trichosporon* in the X8 treatment group was significantly lower than the control group (*p* < 0.05), which indicates that *C. cateniannulata* had an inhibitory effect on *Trichosporon* fungi.

**Figure 8:**
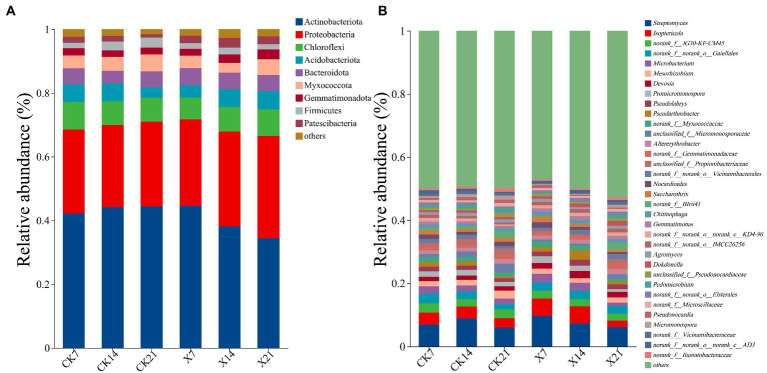
Bacterial flora composition at phylum and genus levels. **(A)** Phylum level. **(B)** Genus level, genera with relative abundance less than 1% are classified as other. X refers to *C. cateniannulata* X8 treatment, CK refers to the control, and the number indicates the time point after inoculation.

Bacterial community species analysis: 1034854 valid sequences were obtained, 37 phyla, 109 classes, 261 orders, 434 families, 827 genera, 1,601 species, and 3,216 OTUs were detected. At the phylum level (Figure 8A), the dominant bacteria were Actinobacteriota (25.72–32.13%), Proteobacteria (6.79–8.65%), Acidobacteriota (4–5.35%). Compared to the control group, the proportion of Actinobacteria in the X8 group showed a downward trend, and Acidobacteria showed an upward trend, indicating that the application of X8 had an inhibitory effect on Actinobacteria and a facilitative effect on Acidobacteria. At the genus level (Figure 8B), *Streptomyces* (5.96–9.65%) was the dominant genus at all time points, and *Lsoptericola* also had a high abundance (2.18–2.95%). In the control group, *Streptomyces* first increased and then decreased over time, while in the X8 group, *Streptomyces* decreased. The abundance of *Lsoptericola* did not change over time in the control group, but showed a significant downward trend in the X8 group.

**Figure 9:**
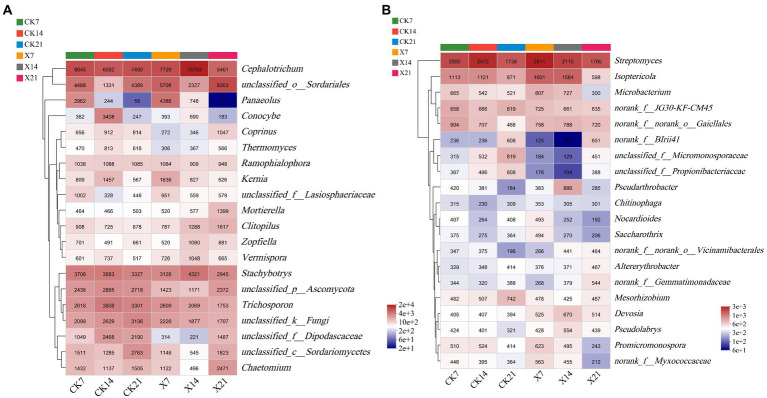
Community heatmap analysis on genus level. **(A)** Fungal community. **(B)** Bacterial community. X refers to *C. cateniannulata* X8 treatment, CK refers to the control, and the number indicates the time point after inoculation.

In the fungal community (Figure 9A), the dominant genera of the two groups were similar on day 7 and 14 but differed on day 21, the control group was *Cephalotrichum*, and the X8 group was unclassified_o_Sordariales. In both groups, the trend of *Panaeolus* was gradually reduced over time, and unclassified_o_Sordariales was increased after the first decrease. In the bacterial community (Figure 9B), the dominant genera of the two groups were the same on day 21, only *Streptomyces*. In the first two periods, the dominant genera in the control group was *Streptomyces*, and the X8 group was *Isoptericola*. *Isoptericola* showed a gradual decrease in both groups.

### 3.6. Correlation between growth indicators and dominant genera

**Figure 10:**
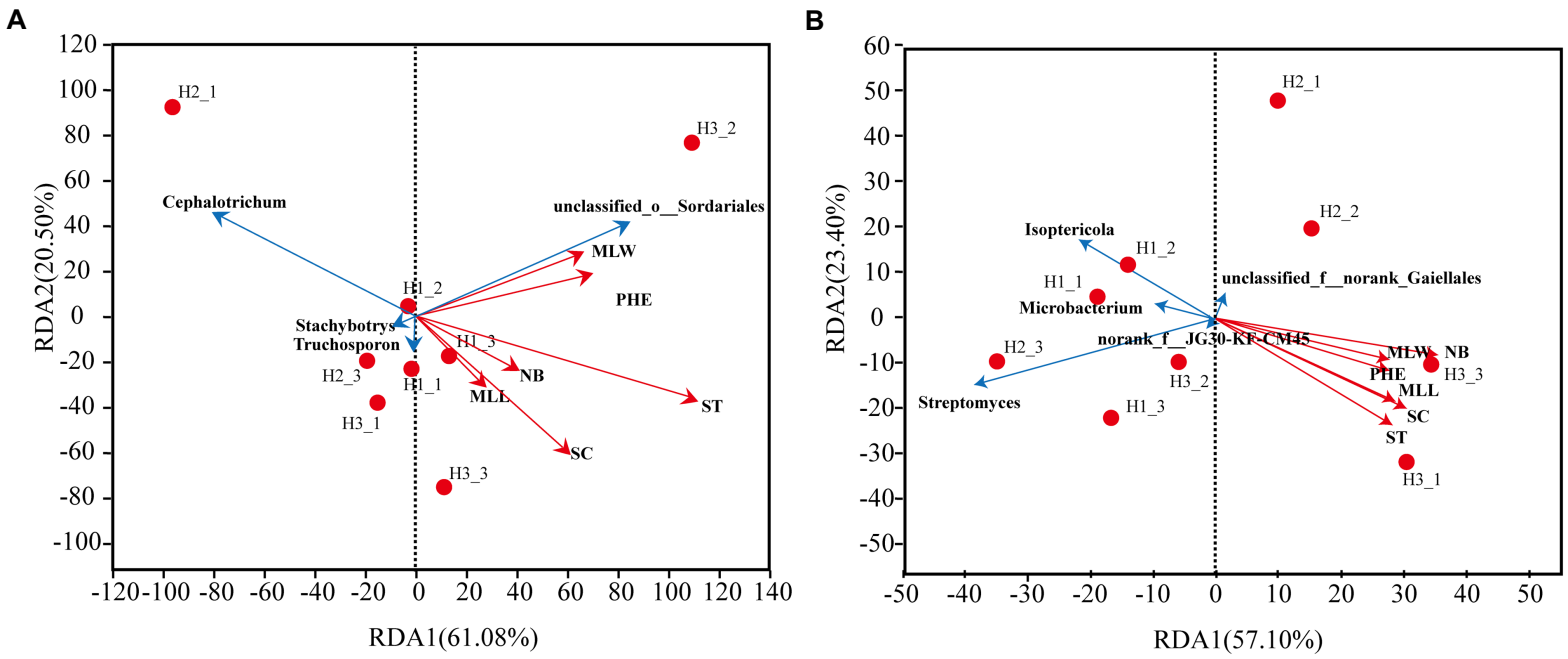
RDA analysis between dominant rhizosphere genera and growth indicators. **(A)** Fungal community. **(B)** Bacterial community. MLL, maximum leaf length; MLW, maximum leaf width; NB, number of leaves; PHE, plant height; ST, stem thickness; SC, stem circumference.

The redundancy analysis (RDA) was used to further study the relationship between growth indicators and dominant genera of rhizosphere microorganisms. In the fungal community (Figure 10A), the accumulation of RDA1 and RDA2 was 61.08 and 20.50%, respectively. The overall explanation for the growth indicators of the fungal community was 61.08%. The stem thickness was the most strongly correlated with the fungal community and accounted for 95.59% of the differences in fungal populations. Unclassified_o_Sordariales were positively correlated to growth indicators. In contrast, *Stachybotrys* and *Cephalotrichum* were negatively correlated to growth indicators. *Trichosporon* with maximum leaf length, stem circumference, leaf number, and stem thickness were positively correlated, and negatively correlated to plant height.

In the bacterial community (Figure 10B), the accumulation of RDA1 and RDA2 was 57.10 and 23.40%, respectively. The overall explanation for the growth indicators of the bacterial community was 57.1%. The number of leaves was most strongly correlated with the bacterial community, accounting for 97.57% of the differences in bacterial populations. *Streptomyces*, *Isoptericola*, and *Microbacterium* were negatively correlated to the growth indicators. The bacterial genus Norank_f_norank_o_Gaiellales with unknown taxonomic status was negatively correlated to stem thickness, stem circumference, maximum leaf length, maximum leaf width, and plant height.

## 4. Discussion

The molecular detection is an effective means to verify that endophytic fungi colonize plants. The target strain *C. cateniannulata* was isolated from tobacco stems, leaves, and roots after inoculation, proving *C. cateniannulata* colonized and transferred in tobacco. Tobacco growth and dry matter accumulation had significantly improved, especially in the middle period after inoculation. These results were in consistent with observation made in other studies (Mantzoukas et al., 2021; Zhang et al., 2022). Those results indicated that some strains of Cordyceps may still retain the characteristics of endosymbiosis with plants in the long-term evolutionary process. Interactions, with entomopathogenic fungi for plants are profitable. The production of plant phytohormones such as auxins, indole acetic acid, gibberellins, and other bioactive compounds may achieve this positive effect (Khan et al., 2015). These compounds are essential in promoting plant growth, such as increasing chlorophyll content, root-shoot length, and biomass production (Kepler et al., 2017). Similar studies have found that entomopathogenic fungi of the genus *Penicillium* can produce substances similar to auxin and cytokinin, which increase plant yields (Toghueo and Boyom, 2020). *Metarhizium anisopliae* can promote root growth by producing indole-3-acetic acid, and enhancing insect virulence after colonization into *Arabidopsis thaliana* (Liao et al., 2017). Therefore, it is possible that *C. cateniannulata* also promotes tobacco growth through production of phytohormones. Furthermore, *C. cateniannulata* may change soil pH, structure, fertility, and oxygen utilization to increase the host’s ability to absorb soil nutrients, thus promoting plant growth (Hong et al., 2017).

Endophytic fungi help the host to absorb water and utilize nutrients, promoting healthy root development, entomopathogenic fungi can also perform this function (Macuphe, 2021). Since entomopathogenic fungi colonize the plant roots, they act as root extensions and probably improve nutrient and water absorption and help plants withstand stress factors (Dara et al., 2017). This symbiosis helps protect the activity of the plant enzyme system, leading to a reduction in MDA content, thus conferring greater stress resistance to the host plant by reducing the damage to the plasma membrane (Card et al., 2021). Entomopathogenic fungi can induce systemic resistance (ISR) by activating jasmonic acid (JA) and ethylene (ET) pathways in the plant, thereby activating the salicylic acid (SA) pathway to acquire resistance (SAR), resulting in the production of plant-related antioxidant enzymes (Doğru, 2021). Related studies have shown that ethylene contributes to plant survival under stress. Further, ethylene levels may increase dramatically and peak under drought stress, to maintain plant water balance by accelerating leaf abscission and senescence and reducing plant transpiration (Waadt et al., 2022). In this study, POD, SOD, and CAT activity were significantly higher in the X8 group than in the control group, indicating that *C. cateniannulata* improved tobacco stress resistance to abiotic environments, protecting cells by SOD, CAT, and POD rapidly removing the free radicals and associated intermediates (Canassa et al., 2019). They can oxidize oxidizing substances produced by respiration, photosynthesis, and auxin to maintain free radicals at an average level and improve plant stress resistance (Radhakrishnan et al., 2013).

Importantly, entomopathogenic fungi can induce the expression of proteins related to plant photosynthesis, energy metabolism, and plant stress response (Bamisile et al., 2020). These proteins induce physiological and biochemical reactions and change nutrient circulation to promote plant growth and enhance resilience (Behie et al., 2017). For example, endophytic fungi can promote proline synthesis in host plants under stress. Moreover, proline may stabilize the structure of biological macromolecules, reduce cell acidity, modulate cell redox potential, and control ROS concentration through coordinating enzyme activity (Segal and Wilson, 2018). All these possibilities may explain the marked increase in resistance to stress in *C. cateniannulata* -colonized tobacco seedlings. At the same time, with the growth of tobacco, its resistance level was also higher, making the ability to scavenge free radicals more vital (Ullah et al., 2019). In this study, *C. cateniannulata* promoted tobacco growth and development. Further, the data provides a beneficial exploration of *C. cateniannulata* in tobacco research and a theoretical basis for the specialized production of endophytic fungi. However, further field studies are required if the yield and quality of tobacco are to be improved using *C. cateniannulata* on a large scale.

The diversity and composition of the rhizosphere microorganisms are closely related to the growth and development of plants (Mendes et al., 2013). The entomopathogenic fungi can promote plant growth by increasing the relative abundance of beneficial microorganisms that are implicated in redox reactions, biological nitrogen fixation, oxidation mineralization, degradation of organic matter, and solubilization (Bever et al., 2012; Tan et al., 2021). Moreover, entomopathogenic fungi can reduce the relative abundance of plant pathogens through competition and the production of secondary metabolites, such as antibiotics (Yan et al., 2019). RDA analyzes revealed that growth indicators were closely related to the rhizosphere microbial communities. *C. cateniannulata* affected plant growth by changing the abundance of specific rhizosphere microbial communities. The abundance of Acidobacteria and Ascomycetes had increased, while the abundance of Actinomycetes, Basidiomycetes and *Trichosporon* had decreased in the soil following *C. cateniannulata* inoculation*. Trichosporon* fungi are pathogenic and cause severe and sometimes fatal infections in immunocompromised patients. Therefore, this study also provides a reference for the application of *C. cateniannulata* in clinical treatment (Matsumoto et al., 2022). The richness of microbial communities was lower than the control group in the early and middle stages after colonization of *C. cateniannulata*, which may be because the growth and reproduction of microorganisms were inhibited when *C. cateniannulata* was initially introduced into the soil, buta new dynamic balance would gradually form over time (Ding et al., 2021). The effect of *C. cateniannulata* on bacterial and fungal communities was significantly different. From the perspective of nutrient competition, the decrease in fungal diversity was beneficial to increase bacterial diversity (Smith et al., 2018).

In conclusion, the results have shown that it is feasible to screen suitable strains of *C. cateniannulata* that can be used in tobacco seedling treatments, thereby resulting in the colonization of target plants. Potential beneficial effects include improved plant growth, increased antioxidant capacity and resistance to microbial phytopathogens. The data bring us a new sight into the ecological relationship among plants, phytopathogens, and entomopathogenic fungi, providing a foundation for future studies of *C. cateniannulata* as an ecological agent.

## Data availability statement

The data presented in the study are deposited in the NCBI GenBank repository, accession number PRJNA938063 (https://www.ncbi.nlm.nih.gov/sra/PRJNA938063), PRJNA938049 (https://www.ncbi.nlm.nih.gov/sra/PRJNA938049).

## Author contributions

LQ and JL designed and performed the experiment and prepared this manuscript. ZZ and ZL performed experiments. YZ and JQ revised the manuscript and language editing. ZY and SX analyzed the data. All authors have read and agreed to the published version of the manuscript.

## Funding

The author(s) declare financial support was received for the research, authorship, and/or publication of this article. This study was supported by the Science and Technology Program of Guizhou Province (2021XM02) and Science and Technology Project of Zunyi Tobacco Company (2022XM18). The funder had the following involvement in the study: experiment design and plant cultivation.

## Conflict of interest

JL, ZZ, and ZL are employed by Guizhou Tobacco Company. The remaining authors declare that the research was conducted in the absence of any commercial or financial relationships that could be construed as a potential conflict of interest.

## Publisher’s note

All claims expressed in this article are solely those of the authors and do not necessarily represent those of their affiliated organizations, or those of the publisher, the editors and the reviewers. Any product that may be evaluated in this article, or claim that may be made by its manufacturer, is not guaranteed or endorsed by the publisher.
