# Corrigendum: Positive effects of *Cordyceps cateniannulata* colonization in tobacco: growth promotion and resistance to abiotic stress

**DOI:** 10.3389/fmicb.2024.1451039

**Published:** 2024-07-04

**Authors:** Lu Qiao, Jing Liu, Zhengxiong Zhou, Zhimo Li, Yeming Zhou, Shaohuan Xu, Zhengkai Yang, Jiaojiao Qu, Xiao Zou

**Affiliations:** ^1^Institute of Fungus Resources, College of Life Sciences, Guizhou University, Guiyang, China; ^2^Zunyi Tobacco Company of Guizhou Province, Zunyi, China; ^3^College of Tea Sciences, Guizhou University, Guiyang, China

**Keywords:** *Cordyceps cateniannulata*, tobacco, endophytic colonization, growing development, rhizosphere microbial diversity

In the published article, there was an error in the Funding statement. The project numbers for the Science and Technology Programs of the Guizhou Province and the Zunyi Tobacco Company were omitted. The correct Funding statement appears below.

