# Supplementary material for: Positive effects of Cordyceps cateniannulata colonization in tobacco: Growth promotion and resistance to abiotic stress
Source: Front Microbiol. 2023 Apr 14;14:1131184. doi: 10.3389/fmicb.2023.1131184 (PMC10140308; doi:10.3389/fmicb.2023.1131184)
Supplement: Supplementary file 1 [file Data_Sheet_1.docx]

Supplementary Material

**The new strategy of** ***C******ordyceps cateniannulata* application in tobacco**

**Lu Qiao ^1^^[[1]](#footnote-1)^†, Jing Liu ^2^^[[2]](#footnote-2)^†, Zhengxiong Zhou ^2^, Zhimo Li ^2^, Yeming Zhou ^1^, Shaohuan Xu ^1^, Zhengkai Yang ^3^, Jiaojiao Qu ^3^, Xiao Zou ^1,^***

*** Correspondence:** Xiao Zou: xzou@gzu.edu.cn

# Supplementary Figures and Tables

## Supplementary Figures

**
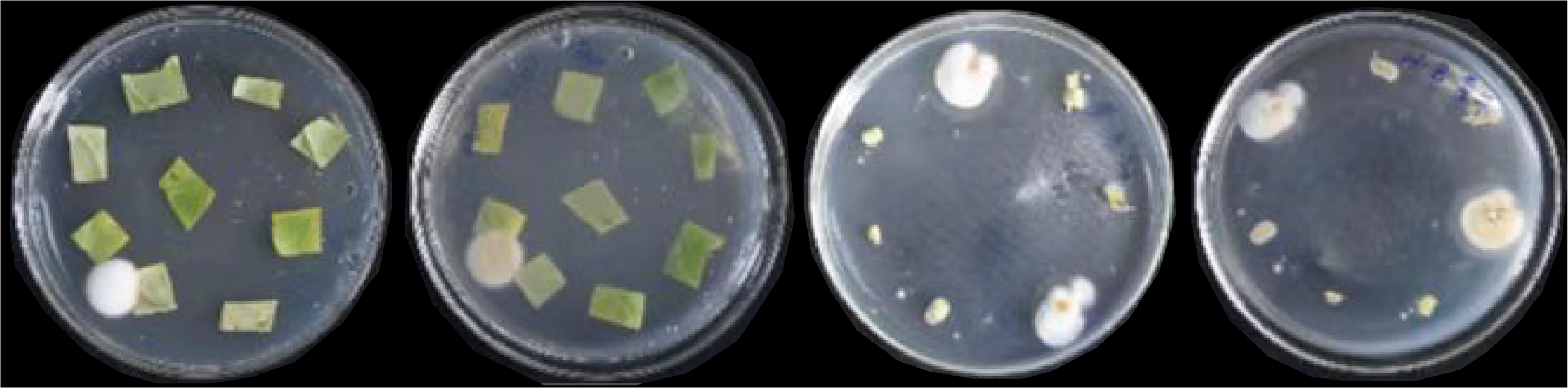
**

**Figure S1.** The colonies induced by tobacco tissue.


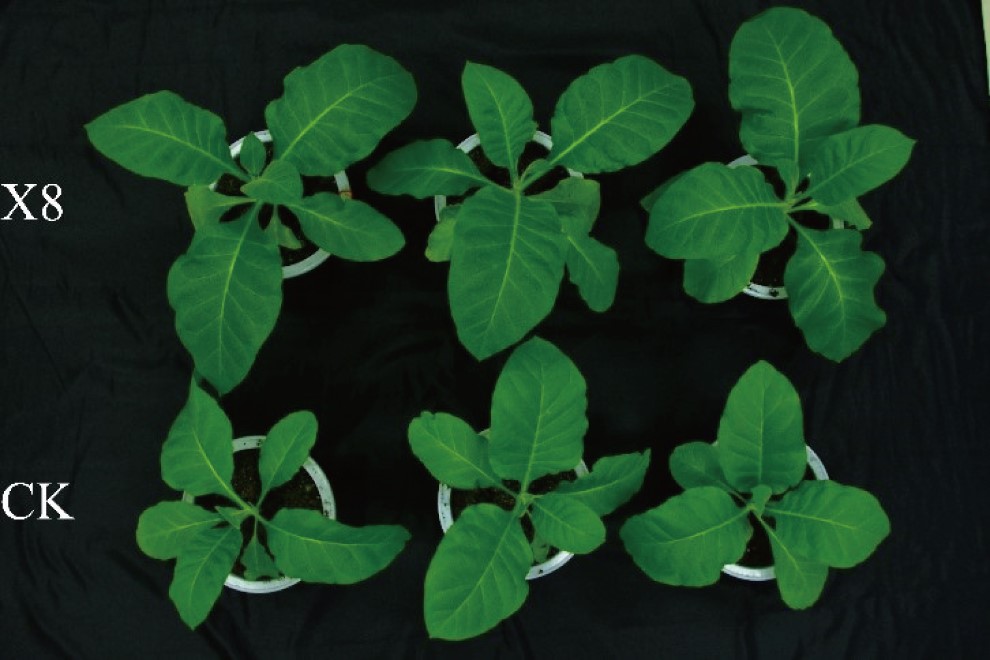


**Figure S2.** Tobacco growth in the X8 and control groups. X8 stands for C. cateniannulata X8, and CK stands for the control.

## Supplementary Tables

## Table S1. Information of *Cordyceps cateniannulata.*

| Strain number | Collection time | Host | Collection site |
| --- | --- | --- | --- |
| GZUIFR04XS8 | 2004 | Lepidoptera nymphs | Kunming West Mountain |

**Table S2.** Method for the determination of peroxidase activity.

| Peroxidase | Method |
| --- | --- |
| POD | Guaiacol method |
| SOD | Nitrogen blue tetrazolium photoreduction method |
| CAT | Hydrogen peroxide method |
| MDA | Thiobarbituric acid method |

**Table S3.** Agronomic indicators of tobacco in the four periods.

|  | Maximum leaf length(cm) | Maximum leaf width(cm) | Number of leaves | Height(cm) | Stem thick(mm) | Stem circumference(cm) | Shoot fresh weight(g) | Shoot dry weight(g) |
| --- | --- | --- | --- | --- | --- | --- | --- | --- |
| CK7 | 22.88±0.61e | 10.75±0.20g | 7.50±0.22e | 6.10±0.18ah | 5.80±0.15f | 2.32±0.08e | 22.34±0.18f | 2.16±0.26g |
| X7 | 25.71±0.96e | 13.30±0.45f | 8.14±0.34e | 8.31±0.53g | 6.30±0.11f | 2.31±0.07e | 26.58±0.09f | 2.32±0.31g |
| CK14 | 28.59±0.95d | 15.76±0.21e | 9.00±0.22d | 10.60±0.55f | 7.16±0.24e | 2.83±0.21d | 49.54±0.21e | 3.72±0.25f |
| X14 | 32.73±0.47c | 16.11±0.20de | 10.43±0.20c | 14.46±0.30e | 7.68±0.14e | 2.96±0.07d | 72.68±0.24d | 6.13±0.54e |
| CK21 | 33.50±2.24c | 15.49±1.04cd | 13.75±0.5b | 20.05±0.9d | 10.61±0.39d | 3.63±0.10c | 97.34±0.37c | 11.73±0.71d |
| X21 | 37.78±3.79b | 18.23±1.07b | 14.00±0b | 22.18±0.91c | 13.09±2.13b | 3.90±0.22b | 131.94±0.55b | 14.02±0.58c |
| CK28 | 37.65±0.79b | 18.85±0.46bc | 16.76±0.46a | 27.57±1.25b | 12.53±0.38c | 4.16±0.64ab | 147.46±1.08b | 18.43±0.93b |
| X28 | 42.83±0.57b | 21.97±0.28a | 17.27±0.78a | 32.52±0.48a | 14.89±0.06a | 4.63±0.19a | 221.42±1.34a | 22.21±0.69a |

CK represents control group, X represents *Cordyceps cateniannulata* group, and the number behind indicates the day of the experiment. Lowercase letters indicate significant differences between different groups (*p* < 0.05).

**Table S4**. Root system development in different periods after transplanting

|  | Length(cm) | Projected Area(cm^2^) | Surface  Area(cm^2^) | Average Diameter(mm) | Root | Tips |
| --- | --- | --- | --- | --- | --- | --- |
|  |  |  |  |  | Volume(cm^3^) |  |
| CK7 | 1638.71±326.32d | 52.94±5.05f | 166.31±15.87f | 0.40±0.07d | 1.48±0.23e | 5800.75±1208.14c |
| X7 | 1903.16±153.60cd | 96.37±7.85e | 285.87±46.09e | 0.48±0.05d | 3.59±0.72de | 8060.00±982.66c |
| CK14 | 2454.78±649.76c | 149.56±19.38d | 412.78±63.07d | 0.39±0.07d | 6.25±1.93d | 8814.00±1081.74c |
| X14 | 3526.50±503.92b | 226.79±33.89c | 712.48±106.48c | 0.66±0.15c | 11.96±4.39c | 13105.20±3015.59bc |
| CK21 | 3193.75±460.59b | 251.20±28.45bc | 789.17±89.38bc | 0.80±0.12b | 15.80±3.39b | 12716.80±1458.63b |
| X21 | 3510.28±703.80b | 268.23±23.08b | 842.67±72.51b | 0.78±0.11b | 16.40±2.31b | 15621.13±4708.14b |
| CK28 | 3434.01±579.54b | 298.67±35.82ab | 959.32±88.31ab | 0.97±0.12ab | 18.65±5.11b | 18973.44±5433.90a |
| X28 | 3893.82±478.87a | 320.19±56.26a | 1043.19±76.21a | 0.12±0.22a | 21.34±4.87a | 22879.34±345.452a |

CK represents control group, X represents *Cordyceps cateniannulata* group, and the number behind indicates the day of the experiment. Lowercase letters indicate significant differences between different groups (*p* < 0.05).

**Table S5.** Alpha diversity of fungal communities and bacterial communities at the OTU level.

|  | Period | | Treatment | Shannon | Ace | Chao | Coverage |
| --- | --- | --- | --- | --- | --- | --- | --- |
| Fungus | | 7 d | CK | 3.749±0.066a | 643.249±27.161ab | 635.742±32.546a | 0.997±0a |
|  | |  | X8 | 3.617±0.036ab | 627.716±13.385ab | 612.949±1.276a | 0.997±0a |
|  | | 14 d | CK | 3.796±0.133a | 583.677±55.115ab | 581.155±71.044a | 0.997±0.001a |
|  | |  | X8 | 3.143±0.046b | 576.938±20.122b | 556.813±34.990a | 0.997±0a |
|  | | 21 d | CK | 3.814±0.350a | 584.143±27.440a | 574.963±58.425a | 0.997±0a |
|  | |  | X8 | 3.569±0.575ab | 615.553±23.530ab | 573.227±37.742a | 0.997±0a |
| Bacteria | | 7 d | CK | 6.082±0.080a | 2440.065±86.412a | 2425.392±37.109a | 0.981±0.001b |
|  | |  | X8 | 5.874±0.017a | 2377.608±101.445a | 2420.855±87.283a | 0.981±0.001b |
|  | | 14 d | CK | 5.982±0.232a | 2410.487±123.729a | 2412.052±96.911a | 0.981±0.001b |
|  | |  | X8 | 5.897±0.280a | 2350.218±147.560ab | 2326.849±175.270ab | 0.982±0.001b |
|  | | 21 d | CK | 5.967±0.109a | 2147.480±70.176b | 2173.645±51.999b | 0.984±0.001a |
|  | |  | X8 | 6.163±0.142a | 2322.119±140.625ab | 2341.596±144.767ab | 0.982±0.002ab |

All the data were presented as mean ± SE (n = 3). SE – standard error of the mean. Different lowercase letters denote significant differences between samples (*p* < 0.05).

The DNA sequence of strain X isolated from plant tissue：

CCCTTCTGTGACCTACCTATCGTTGCTTCGGCGGACTCGCCCCAGCGTCCGGACGGCCCCGCGCCGGCCCGCGACCTGGACCCAGGCGGCCGCCGGAGGCCCTACAACCCTGTATCCATCAGTCTCTCTGAATCCGCCGCAAGGCAAACAAATGAATCAAAACTTTCAACAACGGATCTCTTGGTTCTGGCATCGATGAAGAACGCAGCGAAACGCGATAAGTAATGTGAATTGCAGAATTTAGTGAATCATCGAATCTTTGAACGCACATTGCGCCCGCCAGCATTCTGGCGGGCATGCCTGTTCGAGCGTCATTTCAACCCTCGACGTCCCCTGGGGACGTCGGCCTTGGGGACCGGCAGCACACCGCCGGCCCTGAAATCGAGTGGCGGCCCGTCCGCGGCGACCTCTGCGCAGTACTCCAGCTCGCACCGGGAACCCGACGCGGCCACGCCGTAAAACACCCAACTCTGAACGTTGACCTCGGATCAGGTAGGACTACCCGCTGAACTTAAGCATATCAATAA

1. [↑](#footnote-ref-1)
2. [↑](#footnote-ref-2)
